# Genomic analysis of a rare recurrent *Listeria monocytogenes* prosthetic joint infection indicates a protected niche within biofilm on prosthetic materials

**DOI:** 10.1038/s41598-021-01376-2

**Published:** 2021-11-08

**Authors:** Chloe Hutchins, Lizbeth Sayavedra, Maria Diaz, Puja Gupta, Elizabeth Tissingh, Chiamaka Elumogo, John Nolan, Ian Charles, Ngozi Elumogo, Arjan Narbad

**Affiliations:** 1 Gut Health and Microbes, Quadram Institute Bioscience, Norwich Research Park, Norwich, UK; 2 Microbes in the Food Chain, Quadram Institute Bioscience, Norwich Research Park, Norwich, UK; 3 Norfolk and Norwich University Hospitals NHS Foundation Trust, Norwich, UK; 4 University of East Anglia, Norwich Research Park, Norwich, NR4 7TJ UK

**Keywords:** Biofilms, Clinical microbiology, Policy and public health in microbiology, Health care

## Abstract

*Listeria monocytogenes* is a rare cause of prosthetic joint infections (PJI). In this study, we describe a case of recurrent *L. monocytogenes* infections, 39 months apart, following debridement and retention of a prosthetic hip. Despite numerous studies reporting persistent *L. monocytogenes* in human infections, the genomic and phenotypic changes that clinically relevant strains undergo in the host are poorly understood. Improved knowledge of how PJI occurs is needed to improve the management of prosthetic infections. We used a combination of long- and short-read sequencing to identify any potential genomic differences between two *L. monocytogenes* isolates that occurred over 39-month incubation in the host. The isolates, QI0054 and QI0055, showed three single nucleotide polymorphisms and three insertions or deletions, suggesting that the recurrent infection was caused by the same strain. To identify potential differences in the capacity for persistence of these isolates, their biofilm-forming ability and potential to colonize prosthesis-relevant materials was investigated both in microtitre plates and on prosthetic material titanium, stainless steel 316 and ultra-high molecular weight polyethylene. Whilst the *L. monocytogenes* isolate from the most recent infection (QI0055) was able to form higher biofilm in microtitre plates, this did not lead to an increase in biomass on prosthetic joint materials compared to the initial isolate (QI0054). Both clinical isolates were able to form significantly more biofilm on the two metal prosthetic materials than on the ultra-high molecular weight polyethylene, in contrast to reference strain Scott A. Transcriptomics revealed 41 genes overexpressed in biofilm state and 643 in planktonic state. Moreover, genes with mutations were actively expressed in both isolates. We conclude the isolates are derived from the same strain and hypothesize that *L. monocytogenes* formed biofilm on the prosthetic joint materials, with minimal exposure to stresses, which permitted their survival and growth.

## Introduction

*Listeria monocytogenes* is a facultative intracellular pathogen that can cause serious disease in people who are immunocompromised, pregnant or at extremes of age. Invasive listeriosis and bacteraemia can lead to meningitis, encephalitis, miscarriage and death^1^. *L. monocytogenes* infection is typically associated with foodborne illness. Upon consumption of contaminated food, *L. monocytogenes* can cross the intestinal barrier, spread through the lymphatic and circulatory systems to other organs, and further cross to sites of immune privilege via the blood–brain- and placental barriers^2^. A recent (2017–2018) outbreak of listeriosis in South Africa was associated with the consumption and cross-contamination of a meat-based product. This outbreak caused 1060 known cases^3^ and was described by the World Health Organization as the largest outbreak on record^4^. Whilst such outbreaks can affect many people, particularly if caused by persistence in the food chain of a widely distributed food source, it is also fairly common for sporadic cases of listeriosis to occur^5^.

An uncommon presentation of listeriosis is localised infection^6^ and is usually associated with underlying morbidities. However, cases have been reported in people with no pre-existing health problems, such as veterinarians and farmers exposed to *L. monocytogenes* contaminated bovine abortion material^7^. There is an increasing number of reports of *L. monocytogenes* surgical site infection (SSI), including prosthetic joint infection (PJI). Whilst most SSIs are thought to be nosocomial or from the patient’s skin microbiota, for example *Staphlyococcus*^8^, this is not the case for *L. monocytogenes*. In *Listeria*-associated SSI cases can take years for infection to manifest following primary exposure to this bacterium when the route of acquisition is suspected to be haematogenous^9,10^. Most infections are cleared through prolonged antibiotic treatment, joint debridement or joint replacement^11,12^. Occasionally, chronic infection or recurrence has been reported where there has been an antibiotic treatment failure, and usually where the prosthesis has been retained^13^. However, most cases report successful treatment after replacement of the prosthesis and additional antibiotic therapy^13,14^. Reported follow-ups are within a 24-month period, with few cases lasting beyond 24-months^15,16^, and ‘recurrence’ of the same infection is frequently either assumed or determined from low-resolution typing, rather than confirmed through high-resolution methods such as whole-genome sequencing (WGS)^10,13,14,17^.

In this study, we report a case of recurrent PJI caused by derivatives of the same *L. monocytogenes* strain 39 months after the first infection. We confirm the strain’s ability to form biofilm on prosthetic material surfaces and assess the genomic changes over this period. We propose that prosthetic joint listeriosis can lead to the development of biofilm on the joint, offering *L. monocytogenes* protection against antibiotic treatment and shear stress (e.g. debridement). Biofilm production in the joint might result in asymptomatic persistence of the same strain beyond the time scales previously described for *L. monocytogenes* belonging to Lineage I*,* the most common lineage in clinical cases^18,19^. By comparison, recurrent PJI caused by biofilms of a more common causative organism, *Staphylococcus epidermis,* has been reported to occur within 19 months, on average^20^.

## Materials and methods

### Case characteristics

In 2015, an aspirate was taken from the hip joint of a patient in their 90’s diagnosed with prosthetic joint infection (PJI) as part of clinical care. *L. monocytogenes* grew from this aspirate. Tissue samples were collected intra-operatively, and *L. monocytogenes* (designated QI0054) was isolated from 6 out of 7 tissue samples. Isolate QI0054 was sent to the UK *Listeria* Ref lab, Colindale and identified as serotype 4b, clonal complex 6. The isolates belonged to Lineage I. Debridement and retention of implants procedure was performed. The components (polyethylene liner and femoral head) were exchanged, and a thorough joint washout was carried out. During the first hip replacement, the patient received intravenous amoxicillin and gentamicin before switching to a month-long course of oral amoxicillin 500 mg 3 times per day. The total course of amoxicillin was 16 weeks. 39 months later, the patient was treated with an open washout and debridement. During the procedure, samples were collected from the abscess fluid and *L. monocytogenes* was cultured (designated QI0055) from these. The patient was treated with a course of vancomycin and changed to rifampicin and amoxicillin two days later for a total of 6 weeks. Sample aspirates were collected as part of clinical care. The work was limited to use of previously collected material and no human material was used for this study. All methods were carried out in accordance with relevant guidelines and regulations.

### Cultivation of *Listeria* isolates

An *L. monocytogenes* isolate (“QI0055”) was received from Norfolk and Norwich University Hospital (NNUH) microbiology department and another *L. monocytogenes* isolate, sourced from the same patient at the same infection site 39 months earlier, (“QI0054”) was identified and procured from Public Health England (PHE; Colindale, UK). Both isolates were cultured onto Brilliance *Listeria* agar (ThermoFisher Scientific Oxoid Ltd., Basingstoke, UK) and incubated at 37 °C for 48 h. A single colony of each was used to inoculate 5 mL Brain Heart Infusion (BHI) broth (ThermoFisher) which was incubated at 37 °C with shaking at 180 rpm for ~ 16 h (these conditions were used for liquid culture in all cases unless stated otherwise).

### Growth curve

The growth rate of the two isolates was determined by inoculating 8 replicates of each of the two isolates in 1:10 strength BHI (dBHI) media. Cultures were incubated at 37 °C for 24 h. OD600 was measured every 30 min using a FLUOstar Omega (BMG Labtech GmbH) microplate reader. The growth rate and the doubling time were estimated with the Growthcurver package implemented in R^21^.

### Antibiotic susceptibility testing

The minimum inhibitory concentration (MIC) of 8 antimicrobials was determined by the broth micro-dilution method according to the Clinical and Laboratory Standards Institute (CLSI)^22^. The antibiotic selection panel was selected based on CLSI and EUCAST recommendations as well as additional antibiotics of interest as decided by the clinical infectious disease team^22^. Vancomycin and gentamycin were tested at 0.0312–16 µg/mL, tetracycline at 0.015–8 µg/mL, ampicillin and penicillin at 0.0125–6 µg/mL, gentamicin and meropenem at 0.0078–4 µg/mL and cotrimoxazole at 0.00025–4 µg/mL using lysed horse blood-cation adjusted Mueller Hinton broth (LHB-MHB). Results were collected after incubation at 37 °C for 48 h. Tests were performed in duplicate.

### Biofilm formation of *Listeria* isolates

Both clinical isolates (QI0054 and QI0055) were tested for biofilm-forming ability using a static microtitre assay. For comparison, clinical reference strain Scott A was included as a biofilm control because it is known to consistently form biofilms on plastic and other materials, belongs to the same serotype (4b) and lineage (I) as QI0054/QI0055, and it was originally isolated from a human^23,24^. Overnight liquid cultures were centrifuged for 20 min at 4000 rpm, the supernatant was discarded, and pellets were resuspended in dBHI. Suspensions were then diluted to a concentration of ~ 10^6^ CFU/mL (confirmed by viable counts after plating the culture in dBHI) and 200 µL aliquots added to six wells of a 96-well microtitre plate (96-Well CytoOne^®^ Plate, Non-Treated, flat bottomed; StarLab, Milton Keynes, UK), in triplicate microtitre plates. BHI media without bacterial cells were used as negative controls. Plates were incubated at 37 °C for 48 h, after which the microtitre contents were discarded and the wells were washed once by gently adding 300 µL of sterile deionised water (dH_2_O), with vigorous shaking and blotting in-between the wash step. Microtitre plates were then heated at 50 °C for one hour to fix the biofilm and then stained with 225 µL 0.2% crystal violet (Merck Group, Feltham, UK) for ten minutes. Microtitre plate contents were discarded, the plates blotted onto a paper towel and washed twice with dH_2_O, then left to air dry in MSc Class II cabinets for ~ 15 min. The residual crystal violet, which indicates biomass formation, was solubilised in 30% glacial acetic acid. Absorbance was measured at 590 nm using a FLUOstar Omega plate reader (BMG Labtech). This experiment was conducted in duplicates and absorbance was read in triplicate. The triplicate readings were averaged to account for technical variation in the spectrophotometer reads.

Biofilm-forming ability was determined using a modification of a scoring system described by Stepanović et al.^25^. Briefly, the absorbance readings of the negative controls (i.e. media only) were used to provide a cut-off score (OD_C_), which is three times the standard deviation of these controls. The OD_C_ was then multiplied to provide a scale of biofilm formation as per Table 1. If the mean optical density (OD) of the *L. monocytogenes* isolate being tested was ≤ OD_C_, then the isolate was considered a non-biofilm former and score = 0. If the mean OD of the isolate was ≥ (2 × OD_C_) and < (4 × OD_C_), the isolate was deemed as a moderate biofilm former. Two biological replicates (i.e. separate overnight cultures) were used to calculate the biofilm score for the isolates QI0054 and QI0055, one culture was used to calculate the score for Scott A. The scores are described in Table 1.

**Table 1:** Biofilm scores.

ODc multiplication factor	Biofilm score	Description
1	0	Non-biofilm former
2	1	Low biofilm former
4	2	Mild/modest biofilm former
6	3	Moderate biofilm former
8	4	High biofilm former
16	5	Very high biofilm former

### Biofilm formation of *Listeria* isolates on prosthetic materials

The following materials used in the production of prosthetic joints were investigated: ultra-high molecular weight polyethylene (a plastic further referred to as “polyethylene”), stainless steel 316 and titanium. These were punched into coupons (10 mm diameter, 1 mm depth) which were cleaned with disinfectant (Bioguard, UK), rinsed with deionised water, vortexed in 70% ethanol for ten minutes, and finally sterilised by autoclaving. Materials were procured from Goodfellow Ltd (Cambridge, UK).

For each assay, three sterile coupons were added to individual wells of an untreated 12-well microtitre plate in triplicate, and 3 mL of diluted culture (~ 10^6^ CFU/mL) was added to each well. Negative control wells contained either dBHI alone or dBHI with sterile coupons. The assay was repeated in triplicate (i.e. 9 coupons tested per isolate per material). A single overnight culture was used across all three assays. Microtitre plates were sealed with parafilm. The coupons were incubated at 37 °C under static conditions for five days.

After a 5-day incubation, coupons were washed in ultra-pure water by immersing them for ten seconds, twice, to remove loosely attached cells. Coupons were then added to 3 mL PBS with 5 sterile glass beads (2 mm) and vortexed for 10 min. The resulting suspension was serially diluted and 50 µL aliquots were plated onto BHI agar plates and incubated at 37 °C for 48 h.

### Biofilm analysis

Bacterial counts were calculated as CFU/coupon. The minimum CFU/coupon that we could detect was 60. Planktonic CFU/mL counts grouped by material were compared using a one-way analysis of variance (ANOVA). Planktonic CFU/mL were log_(10)_ transformed to report differences from the starting inocula. Statistical analyses were performed in R and graphs produced using Graphpad Prism (v.7). Mean OD values were compared using a one-way ANOVA. For material surface biofilm counts, we did not consider values that were 0 as these were caused by the minimum CFU counts that could be measured per coupon (i.e. below the detection limit of the assay and not true zero). A linear model was created with the lm function in R (CFU/coupon ~ strain + material)^26^. Pairwise comparisons were done using the emmeans package^27^ considering the effect of the strain and the material pairs (emmeans (model, ~ strain + material)). Only comparisons between the same material or the same strain were considered. We considered that results were significant when *p* value < 0.05.

### Transcriptomics

RNA was extracted from QI0054 and QI0055 in planktonic or in biofilm form. For the biofilm state, 4 coupons of each of the three materials, polyethylene, stainless steel 316 and titanium, were incubated with the *L. monocytogenes* strains as described above (see “Biofilm formation of *Listeria* isolates”) with the following modifications: samples were fixed with 1 mL of RNAprotect (Qiagen) and stored at 4 °C overnight before detaching the cells from the coupons as described above. Total RNA was purified with the RNeasy Mini Kit (Qiagen) according to manufacturer instructions. Briefly, cells were enzymatically and mechanically lysed using lysozyme, proteinase K and lysing matrix E beads (MP Biomedicals, UK). RNA was purified using the RNeasy Mini Kit protocol with the on-column DNase digestion step.

Total RNA was sent to Genewiz (Germany) for library preparation and sequencing. rRNA was depleted with the NEBNext rRNA depletion kit (bacteria) and libraries were prepared with the NEBNext ultra II RNA RNA library prep kit for Illumina according to the manufacturer’s instructions (NEB, MA, USA). Samples were sequenced at a depth of ~ 20 million 2 × 150 bp reads per sample on an Illumina NovaSeq 6000 instrument. For differential expression analyses, reads were processed as described previously^28^. Briefly, adapters and ribosomal reads were removed, and reads were quality trimmed to a minimum quality of 10 using bbduk (v.37.02). Clean reads were mapped to the genome of QI0054 with a minimum identity of 95% with all ambiguous reads reported using bbmap. Some of the reads from the biofilm samples showed mapping to other organisms. This could be explained by the low RNA yield from biofilm samples, due to the low input biomass (0.2–1.3 ng/µL). Any potential contamination during RNA extraction, library preparation or sequencing is potentially detected when using a low RNA yield, but the high similarity threshold of the mapping accounted for this. The number of reads per coding sequence was determined using featureCounts (v.2.0)^29^. Differentially expressed genes were detected with edgeR with the count matrix normalized using TMM^30,31^.

### Whole-genome sequencing and assembly

Individual colonies of strains QI0054 and QI0055 (n = 1) were used to inoculate 1 mL BHI and incubated as described above. Genomic DNA was extracted from the resulting cultures using GenElute™ Bacterial Genomic DNA Kit (Merck, UK). DNA was used for library preparation using Illumina Nextera low input tagmentation (Illumina, UK) and whole-genome sequencing using a NextSeq500 instrument (Quadram Institute Bioscience, UK). To close the genomes, DNA was sent to Novogene (China) for long-read sequencing with PacBio (Novogene, China). Short reads were quality trimmed with a minimum quality of 2 and adapters removed using bbduk (v.37.02) (trimq = 2 ftl = 10 qtrim = rl). The quality of the cleaned reads was inspected using FastQC. Hybrid assemblies for strains QI055 and QI0055 were reconstructed using Unicycler with standard settings (v.0.4.7)^32^, which resulted in one single scaffold for each isolate. Unicycler used the read error correction module from SPAdes (v.3.12.0) before the initial round of assembly and polished the assembly with Racon (v.1.3.1) and Pilon (v1.22)^33,34^. The assembly graphs were inspected with Bandage which revealed that the end of the scaffolds were connected to themselves suggesting a closed circular genome. Genome completeness was assessed with CheckM (v.1.1.3) using 325 marker genes (Bacilli UID285)^35^.

### Phylogenomic reconstruction, SNP counts, and mutation rate estimation

To identify the lineage of QI0054 and QI0055, we retrieved 260 high-quality genomes covering the five major *L. monocytogenes* lineages from the Patric database (accessed on 16.01.2020)^36^. Except for the representative of lineage IV, all were assembled in less than five scaffolds. The quality of these genomes was calculated using checkM (v.1.1.12)^35^ and quast (v.5.0.2)^37^. All genomes had a completeness higher than 92.86% and a contamination less than 5.19%. Genomes were annotated with Prokka (v.1.14)^38^. The genes shared by more than 99% of strains (core genes) were calculated using Roary (v.3.1.3)^39^, which resulted in 2309 core genes. We extracted the variable positions of the aligned core genes using snp-sites (v.2.5.1)^40^, which resulted in 36,056 alignment positions. The SNP alignment was used to reconstruct a tree using RaxML (v.8.2.12)^41^ with a function that corrects for ascertainment bias from SNP data (-m ASC_GTRGAMMA –asc-corr = Lewis). The best scoring tree was visualized with iTol^42^.

To search for the closest epidemiological relatives, we used BacWGSTdb 2.0^43^. The closest four isolates based on the SNP strategy from BacWGSTdb were used to search for the closest genomes at NCBI’s Isolate Browser (ncbi.nlm.nih.gov/pathogens/isolates/) using a SNP distance of 14. This search resulted in 63 genomes belonging to the SNP cluster PDS000024682.110. SNPs between the closest relative strain genomes and our clinical isolates were estimated with three different methods: (i) Our first method used Snippy (v.4.2.1) with QI0054 used as reference^44,45^. The number of SNPs for genomes with fewer than 50 SNPs was refined using the reads trimmed to a minimum quality of 20 as a small variation in the number of SNPs is expected due to potential miss-assemblies in the genomes used for comparison; ii) Our second method used a de novo analysis of the core genome with Roary, which included only the closest epidemiological strains to QI0054, and *L. monocytogenes* EGD-e (NC_003210) (core genome = 2437 genes). Strain EGD-e was included in the analysis to identify the ortholog genes of the core genes defined by Moura et al.^46^ (for method (iii)). Recombinant regions were removed with Gubbins (v.2.3.4)^47^, variable monomorphic positions (-b option) were extracted with snp-sites (v.2.5.1), and the number of SNPs was estimated with snp-dists (v.0.7.0)^40^; (iii) Finally, we extracted the genes defined by Moura et al.^46^ for core-genome multilocus sequence typing (cgMLST = 1748 genes). Regions of the alignment with recombination were removed and variable positions were extracted as described above. The mutation rate was estimated with BactDating^48^ using the core genes from method (iii).

## Results

### Genomic comparison of initial and recurrent *L. monocytogenes* isolates

We assembled closed genomes of the two isolates obtained from the hip joint of the same patient (QI0054 and QI0055) using hybrid assemblies with short- and long-read sequencing. The two genomes were assembled in single scaffolds of 3.034 Mb, had a 37.96% GC content, and were estimated to be 99.45% complete. The genome size was within the expected range of other *L. monocytogenes* isolates (2.77–3.1 Mb). Plasmids or phage sequences were not detected.

We estimated the number of SNPs and InDels between QI0054 and QI0055 to determine if they had the same origin (Table 2). There were only three InDels that caused frameshifts and three missense SNPs differences. The InDels were found in a GTP pyrophosphokinase (*relA*), maltose phosphorylase (*malP*), and a helix-turn-helix domain-containing protein. SNPs were found on the genes encoding a glycosyl hydrolase of the family 31, rod shape-determining protein MreB, and virulence regulator PrfA.

**Table 2:** SNP and InDels between QI0054 and QI0055 detected by snippy (v.4.2.1).

Position	Type	Ref	Alt	Evidence	Nt	AA	Effect	Locus tag (LR999861/NP_463670)	Product
228,590	snp	C	G	G:53 C:0	707/3303	236/1100	Missense variant c.707C>G p.Ala236Gly	QI0054_263/lmo0182	Glycosyl hydrolase, family 31
249,573	snp	C	T	T:67 C:0	385/714	129/237	Missense variant c.385G>A p.Ala129Thr	QI0054_281/lmo0200	Virulence regulatory factor PrfA
1,639,252	del	ACTTG	A	A:64 ACTTG:0	2044/2217	681/738	Frameshift variant c.2041_2044delCAAG p.Gln681fs	QI0054_1707/lmo1523	Guanosine-3′,5′-bis(diphosphate) 3′-pyrophosphohydrolase (EC/GTP pyrophosphokinase (EC, (p)ppGpp synthetase II
1,665,380	snp	G	A	A:106 G:0	343/1014	115/337	Missense variant c.343C>T p.Arg115Cys	QI0054_1732/lmo01548	Rod shape-determining protein MreB
2,293,239	del	AT	A	A:28 AT:0	221/2262	74/753	Frameshift variant c.221delA p.Asn74fs	QI0054_2308/lmo2121	Maltose phosphorylase
2,773,205	del	CA	C	C:55 CA:1	177/213	59/70	Frameshift variant c.177delT p.Phe59fs	QI0054_2785	FIG00774055: hypothetical protein

The genome of QI0054 was used as reference (accession LR999861). The genome NP_463670 is commonly used as a reference *L. monocytogenes* genome, so the ortholog reference gene is included when applicable. Nt = nucleotide; AA = amino acid; fs = frame shift; c = change.

### SNP analysis and mutation rate of *L. monocytogenes* closest relatives

**Figure 1:**
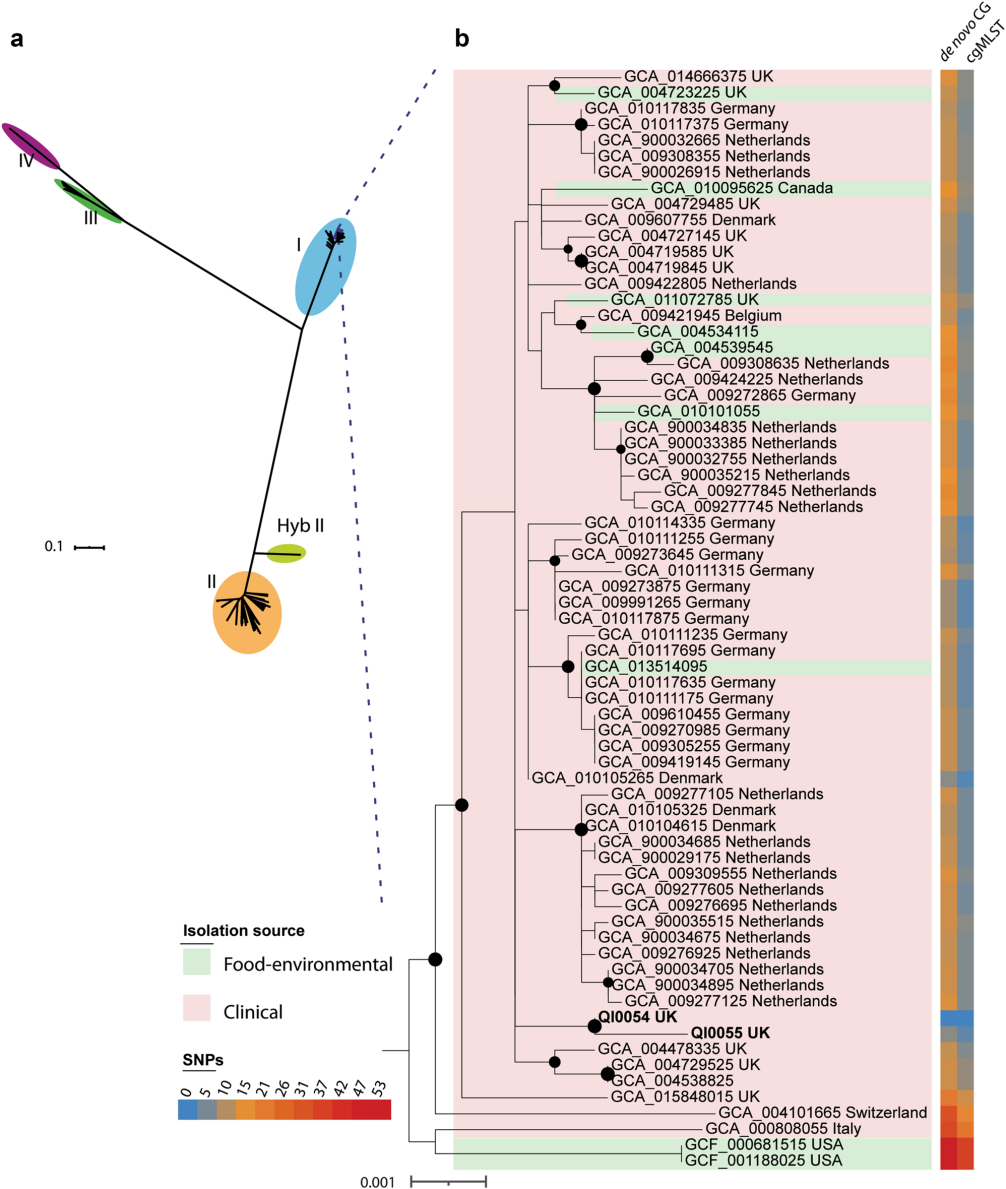
Closest relatives of *L. monocytogenes* QI0054 and QI0055 strains, and number of SNP changes. **a** Phylogenetic reconstruction of *Listeria monocytogenes* isolates determined by maximum likelihood. The tree was reconstructed using the variable positions of the 2309 core genes found among 261 *Listeria* genomes. The scale bar represents the mean number of nucleotide substitutions per site. **b** SNP phylogenetic reconstruction with the closest epidemiological relatives and the number of mutations detected using de novo calculation of the core genome (method II) or the cgMLST genes^46^ (method III, see “Materials and Methods” section).

QI0054 and QI0055 clustered with members of lineage I, based on a well-supported phylogenomic tree of *L. monocytogenes* with 260 representatives of the four major known lineages and the hybrid sub-lineage II (Fig. 1a)^49^. Most of the closest epidemiological-linked strains were of clinical origin (Fig. 1b). These strains were found using BacWGSTdb 2.0^43^ and NCBI’s isolate browser. Our SNP phylogeny could not resolve the branching pattern of the closest subclade containing QI0054 and QI0055 and resulted in a polytomy. Thus, to identify the closest relatives, we estimated the number of SNPs between the closest *L. monocytogenes* strains using three methods: (I) SNP calls using as reference genome QI0054 with snippy; (II) de novo calculation of the core genome and quantification of variable positions after removing potential recombination, and; (III) quantification of the variable positions considering only the genes defined as cgMLST by Moura (see Supplementary Table S1).

58 isolates had less than 7 variable positions compared to the genome of QI0054 in the cgMLST genes defined by Moura et al.^46^. These same 58 isolates ranged between 7 and 16 SNPs using method (II) and ranged between 6 and 368 SNPs using method (I) (Supplementary Table S1). Interestingly, all the 58 isolates were of European origin.

The mutation rate of the closest epidemiological strains to QI0054 was ~ 9.5 × 10^–7^ substitutions per site per year, as inferred from the core genome (1.5 substitutions per 1.58 Mb cgMLST). Root to tip distances were significantly associated with the year of isolation for this sub-clade (*p* = 9 × 10^–3^, BactDating). This mutation rate is higher compared to mutation rates previously estimated for *L. monocytogenes* obtained from food-production factories (1.15 × 10^–7^ substitutions per year)^50^ or rates estimated for the most prevalent sublineage SL1 (2.6 × 10^–7^) or sublineage SL9 (2.4 × 10^–7^)^46^.

### Phenotypic characterisation of *L. monocytogenes* isolates

#### Antibiotic susceptibility

The original and recurrent *L. monocytogenes* isolates did not show a change in antibiotic susceptibility. Minimum inhibitory concentrations (MIC) revealed that QI0054 and QI0055 were equally sensitive to the following antibiotics (µg/mL): ampicillin, > 6.08; meropenem, 0.25; cotrimoxazole, 0.000025; penicillin, 0.38; vancomycin, 1; gentamicin, 0.5; tetracycline, 1; ciprofloxacin, 1.

#### Growth rate and biofilm formation

Some bacteria accumulate mutations that cause them to grow slower and can provide them with an escape mechanism for antibiotic stress^51^. Thus, we tested the growth rate of QI0054 and QI0055 isolates. Both isolates showed a similar growth rate (r = 0.3) and doubling time (2.1–2.2 h) in dBHI (Supplementary Fig. S1), suggesting no alteration in the growth behaviour.

**Figure 2:**
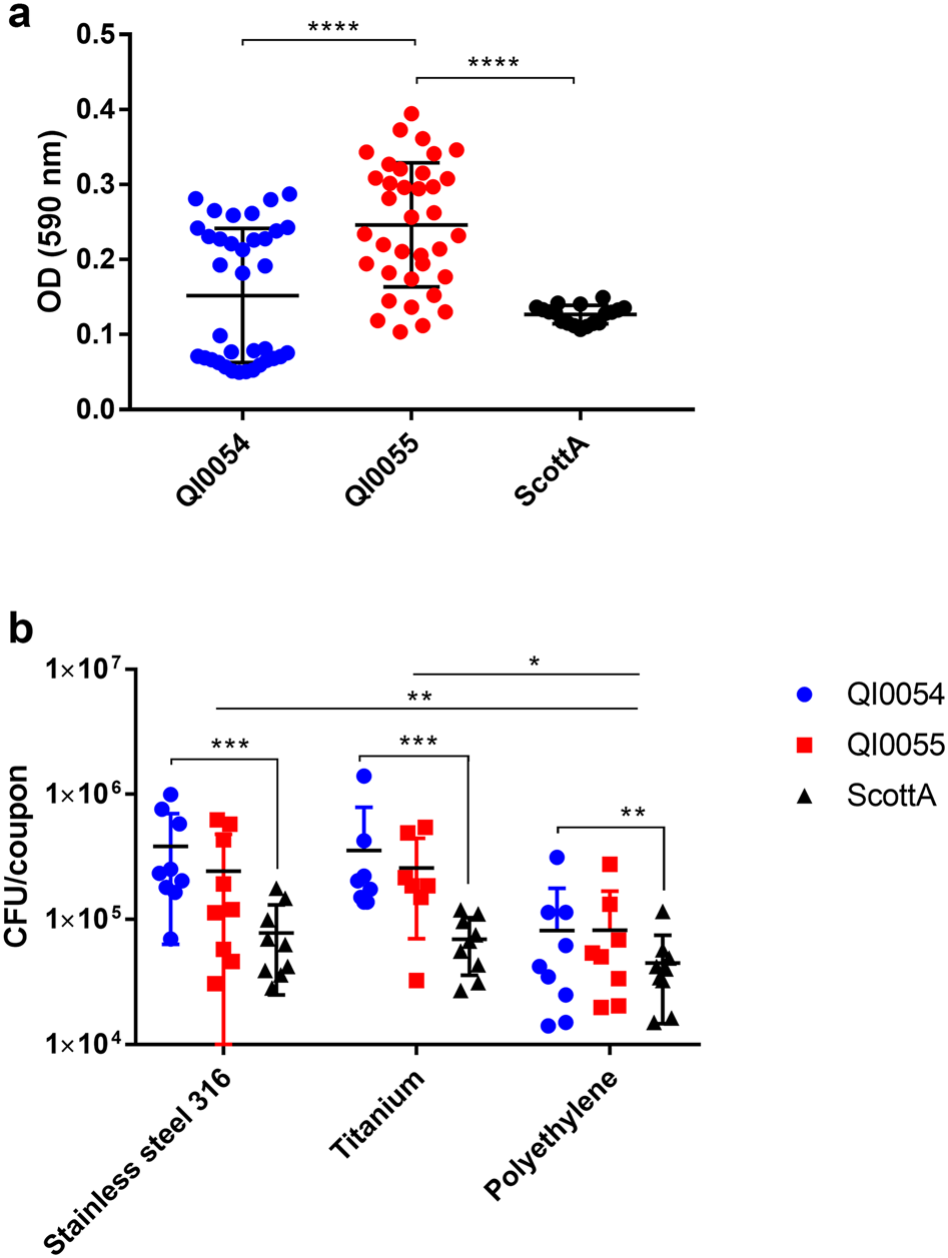
(**a**) Average (± SD) OD values of *L. monocytogenes* strains tested for biofilm-forming ability. Clinical isolates QI0054, QI0055 and reference strain Scott A. Samples were measured in 2 96-well plates with 18 replicates each (n = 36). (**b**) Average (± SD) CFU/coupon of *L. monocytogenes* recovered from prosthesis-relevant materials; n = 9. The three materials tested were stainless steel 316, titanium and ultra-high molecular weight polyethylene (“polyethylene”). ***p value < 0.01; **p value (0.01–0.049); *p value (0.05–0.09).

Strains QI0054 and QI0055 were able to form low-moderate biofilm using standard static microtitre biofilm assays. We compared these clinical isolates with clinical reference strain Scott A. There was a small but significant difference (*p* < 0.0001) between biofilm formation in QI0054 and QI0055 (Fig. 2), which both scored 1–2. Scott A strain had a score of 1.

#### Biofilm formation and transcriptional activity on prosthetic materials

The materials we used to test biofilm formation did not influence the planktonic growth of QI0054, QI0055, or of the reference strain Scott A. For 5 days, planktonic bacterial numbers increased for all three cultures, with mean log_10_ increase from the starting inoculum of 1.56 for QI0054, 1.13 for QI0055 and 1.22 for Scott A. No significant difference in planktonic growth was observed among the different prosthetic materials tested (*p* = 0.0685; one-way ANOVA).

We then tested whether there was a difference in biofilm formation on material surfaces considering the biofilm formed by strains QI0054, QI0055 and Scott A (Fig. 2b). The *L. monocytogenes* strains formed significantly higher biofilm on stainless steel compared to polyethylene (*p* = 0.015, *t* test), and, although not significant, higher biofilm in titanium compared to polyethylene (*p* = 0.08, *t* test). The median *L. monocytogenes* recovered from biofilm formed on material surfaces (CFU/coupon) was 4.2 × 10^4^ on polyethylene, 1.4 × 10^5^ on titanium, and 1.5 × 10^5^ on stainless steel (Fig. 2b). Moreover, QI0054 formed higher biofilms compared to the reference strain ScottA in all three materials tested (*p* value = 0.0267, *t* test, Fig. 2b). These results suggest that, in this case, the polyethylene component of the prosthetic hip joint could have been less facilitating of biofilm than the metal components. However, different clinical strains may have different affinities for biofilm formation in the joint environment.

We used whole transcriptome analysis of QI0054 and QI0055 in planktonic and biofilm state to investigate the genes related to biofilm formation on these isolates (n = 2 extractions per strain per condition). 41 genes were overexpressed when our isolates were in biofilm state, while 643 were overexpressed in planktonic state (Supplementary Table S2). Among the genes overexpressed in planktonic state, we found genes previously identified to be involved in biofilm formation and motility. These genes included an internalin A (lmo0433), a flagellin protein (lmo0690), an attachment-related lipoprotein (lmo1068), and lipoprotein assembly (lmo2482) (See Supplementary Table S2)^52,53^. Among the genes overexpressed in biofilm state, we identified genes related to amino acid and nucleotide synthesis, an internalin-like protein (lmo0549), quorum-quenching lactonase (lmo1614), and plasmid replication DNA element (lmo2221). The latter three genes were previously identified to be involved in biofilm formation of *L. monocytogenes* in dBHI^54^.

Finally, we investigated whether the genes containing SNPs or InDels were expressed in our isolates. All six genes were found expressed by both, QI0054 and QI0055 in planktonic state (Supplementary Table S2). The expression of hypothetical protein QI0054_2785 was not detected in biofilm state, and *relA* (lmo1523) was found overexpressed in planktonic state. We did not detect any differentially expressed genes between QI0054 and QI0055 in biofilm state.

## Discussion

*L. monocytogenes* is an organism commonly associated with foodborne illness, although it has also been infrequently associated with bone and joint infections, particularly where there are prosthetic implants^13^. Recurrent and chronic infections have been reported mostly in cases where prosthesis has been retained. However, the majority of reported infections recurred, or were successfully treated within, 2 years since the initial detection of *L. monocytogenes* infection^15^.

In this study we report two isolates, only three SNPs and three InDels apart, obtained from the same patient with 39 months between isolation, strongly suggesting that the reservoir of reinfection was biofilm on the prosthetic joint materials. The number of genomic differences between our isolates contrasts with two *L. monocytogenes* isolates belonging to lineage II reported on a recurrent hip infection five years apart (strains: N843_15 and N843_10; 22 SNPs and 44 InDels, but only seven cgMLST allelic differences)^16^. The cut-off for distinguishing *L. monocytogenes* outbreaks lies between 7 to 12 SNP differences in lineage I and II^55,56^. Additionally, according to Moura et al.^46^ most isolates with less than seven allelic mismatches in the cgMLST originate from single outbreaks. The level of genomic differences reported in the five-year case suggests that these isolates may have either i) been caused by different infection events or ii) an increased mutation rate, potentially triggered by to antibiotic treatment or other stressors within the host environment^13^. Higher mutation rates have been observed in cases of persistent infection, particularly where antibiotic treatment has not been successful in clearing infection^57^.

The analysis of the mutation rate in our isolates and the closest epidemiological strains showed a higher mutation rate among these strains compared to *L. monocytogenes* from environmental sources and other strains from lineage I (~ 3.8-fold more substitutions). The high mutation rate of our isolates could suggest that this sublineage is highly divergent compared to other *L. monocytogenes* belonging to Lineage I. A possible explanation could be that isolates from this subclade came from clinical cases and therefore may have been exposed to higher environmental stressors within the host. Among the closest epidemiological relatives, we detected 58 isolates with less than seven allelic differences in the cgMLST of European origin, including the UK, Denmark, Germany, and the Netherlands. Based on the number of allelic differences, these isolates could have originated from the same source. However, we do not have enough evidence or data to trace their origin.

Interestingly, in both recurrent *L. monocytogenes* cases, missense mutations in the gene of the virulence regulator *prfA* (lmo0200) occurred. PrfA is the master regulator of virulence in *L. monocytogenes*. Deleterious mutations in this gene tend to be eliminated from the population since these mutations could potentially attenuate the virulence capacity of the strain^58^. PrfA plays an important role in the biofilm-forming ability of *L. monocytogenes* and has been previously shown to affect the expression of 175 genes during biofilm formation compared to a wild type and a *prfA* deletion mutant^59,60^. The missense mutation detected in *prfA* from QI0055 did not significantly affect the expression profile when in biofilm state, which suggests that the biofilm-related activity of *prfA* remained intact. SNPs found in QI0055 compared to QI0054 were also present in genes associated with the cell wall structure, which potentially influence motility and surface adhesion, as well as biofilm dispersal^61,62^. One of the genes identified to have an InDel, *relA* (lmo1523), is reported to have various roles in virulence and influencing the *luxS* quorum-sensing mechanism, which is also involved in the biofilm life cycle^63–65^. The genomic changes, therefore, suggest that long-term infection impacts genes related to biofilm formation and quorum-sensing mechanisms.

### Phenotypic traits

The biofilm-forming capacity of the QI0055 recurrent isolate was higher compared to the original isolate QI0054 in microtitres plates (Fig. 2a), but the opposite trend was observed on two of the prosthesis-relevant materials. The recurrent *L. monocytogenes* (Lineage II) case reported by Muchaamba et al.^16^ showed that the more recently isolated strain’s biofilm capacity was lower than the original infection strain. This is consistent with our observation of a lower biofilm capacity on relevant materials. By comparison to the case described by Muchaamba et al.^16^, QI0054 and QI0055 (Lineage I) had fewer genomic and phenotypic changes and did not change their antibiotic profile. Biofilm formation will likely depend on the material surface and the surrounding fluid, but our results suggest that high biofilm-forming ability does not necessarily contribute to the long term persistence of *L. monocytogenes* on prosthetic joints. Regardless of the extent of biofilm formation by *L. monocytogenes* (considered in our study as a biofilm “score”), the ability to form biofilm de facto allowed for the long-term propagation of similar biomass on prosthetic material surfaces.

The environmental cue that led to the re-emergence of the persistent strain after such a lengthy dormant period is unknown. *L. monocytogenes* usually causes bacteraemia with sepsis, and although blood cultures were negative, it is plausible that the patient had an episode of bacteraemia at some point with subsequent seeding to the prosthetic material. In our case, debridement and retainment of the joint, along with antibiotic treatment, led to asymptomatic persistence for 39 months, followed by a resurgence of the same strain. *L. monocytogenes’* ability to persist in cell vacuoles could have also contributed to asymptomatic persistence and evasion of antibiotic therapy^66^.

The material surfaces investigated, titanium, stainless steel 316 and polyethylene, have been shown to support biofilm in previous studies. *L. monocytogenes* is well known to form biofilm on titanium and stainless steel 316 surfaces. *L. monocytogenes* has been isolated from a similar material to ultra-high molecular weight polyethylene in the food production industry^67^. However, to the best of our knowledge, this is the first report that shows the ability of *L. monocytogenes* to form biofilms on ultra-high molecular weight polyethylene used for prosthetic implants. Polyethylene has been shown to support biofilms of two clinically significant organisms, *Pseudomonas aeruginosa* and *Staphylococcus aureus*, and after antibiotic treatment, polyethylene showed no viable cells for these organisms from the material surfaces after a 24-h exposure^68^. However, the same study demonstrated that viable cells could be recovered from the antibiotic-treated biofilms after 72-h of exposure, implying a state of protection or dormancy of biofilm cells. This highlights the persistent nature of biofilm after seemingly successful treatment. Currently, efforts to improve the antimicrobial nature of these surfaces through nanoparticle and photocatalytic particle coatings are being developed with promising results against *S. aureus* infection in animal models^69,70^.

All prosthesis-relevant materials tested in this study were able to support *L. monocytogenes* biofilm formation. Whilst the ultra-high molecular weight polyethylene tested led to slightly lower levels of biofilm than the metals titanium and stainless steel, the number of biofilm cells recovered was relatively high on all three materials, indicating it was unlikely that an individual material acted as a primary reservoir of *L. monocytogenes*. However, a limitation of this study is that the experiments could not be compared to biofilm formation in synovial fluid or on bone cement. We confirmed that *L. monocytogenes* recovered in 2015 and 2019 from the prosthetic site were a derivative of the same strain, but the most recent isolate had mutations in biofilm dispersal- and virulence-relevant genes. This case highlights the risk of recurrent infection if prosthesis infected by *L. monocytogenes* is retained, allowing silent persistence of the same strain in the form of biofilm for periods of more than 3 years. This case, along with other recurrent or persistent *L. monocytogenes* PJI^15,16^ support extended clinical monitoring after treatment (> 24 months).

## Supplementary Information

Supplementary Figure S1.

Supplementary Table S1.

Supplementary Table S2.

## Data Availability

Genome assemblies and raw sequencing data was submitted to the European Nucleotide Archive (http://www.ebi.ac.uk/ena/data/view/) under the Accession Number PRJEB40663. Transcriptome sequencing data can be accessed under the Accession Number PRJEB46991. Strains QI0054 and QI0055 are available upon request from the QIB culture collection.

## Abbreviations

SNP: Single nucleotide polymorphism
InDels: Insertions or deletions
WGS: Whole genome sequencing
SSI: Surgical site infection
PJI: Prosthetic joint infection
