# Supplementary material for: Genomic analysis of a rare recurrent Listeria monocytogenes prosthetic joint infection indicates a protected niche within biofilm on prosthetic materials
Source: Sci Rep. 2021 Nov 8;11:21864. doi: 10.1038/s41598-021-01376-2 (PMC8575960; doi:10.1038/s41598-021-01376-2)
Supplement: Supplementary file 1 — Supplementary Figure S1. [file 41598_2021_1376_MOESM1_ESM.docx]

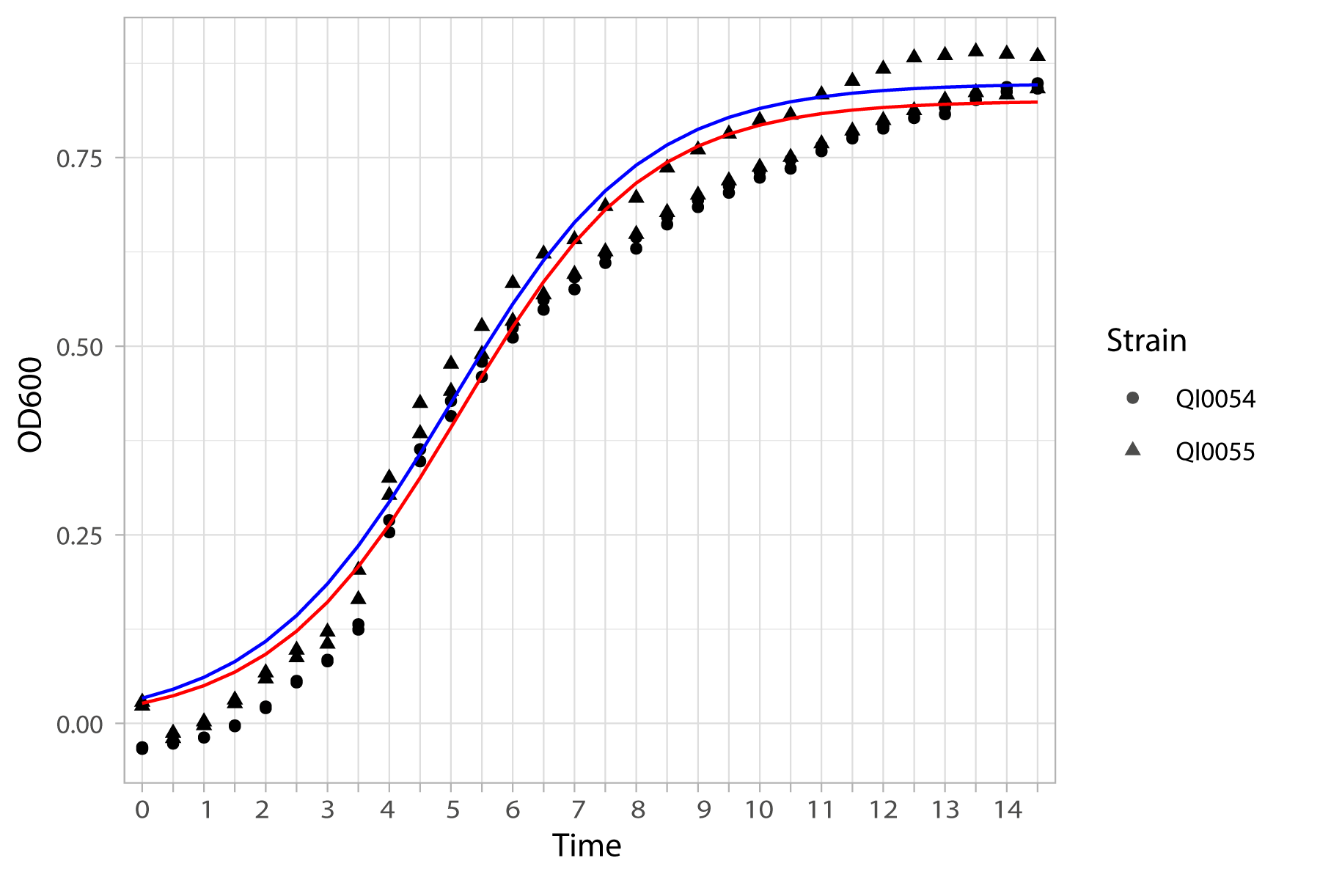


**Supplementary Figure 1:** Growth curve of isolates QI0054 and QI0055. The growth curve estimates determined by the Growthcurver package are shown (QI0055 in blue; QI0054 in red).
